# Multifaceted roles of RNA polymerase IV in plant growth and development

**DOI:** 10.1093/jxb/eraa346

**Published:** 2020-09-24

**Authors:** Shuai Zhang, Xiao-Qing Wu, Hui-Ting Xie, Shan-Shan Zhao, Jian-Guo Wu

**Affiliations:** Vector-borne Virus Research Center, State Key Laboratory of Ecological Pest Control for Fujian and Taiwan Crops, Fujian Province Key Laboratory of Plant Virology, Fujian Agriculture and Forestry University, Fuzhou, China

**Keywords:** Arabidopsis, plant growth and development, Pol IV, rice, RNA-directed DNA methylation, siRNA biogenesis

## Abstract

We discuss the latest findings on RNA polymerase IV (Pol IV) in plant growth and development, providing new insights and expanding on new ideas for further, more in-depth research on Pol IV.

RNA-directed DNA methylation (RdDM) is a small RNA-mediated epigenetic process in plants. The biogenesis of small RNAs and initiation of RdDM rely on complex transcriptional machineries, including two plant-specific RNA polymerases (Pol IV and Pol V) and other auxiliary proteins. Pol IV is known to play a critical role in generating 24-nt siRNAs in the RdDM pathway, and is involved in *Capsella* pollen development, rice tillering, and rice resistance to viruses. Here, we discuss the most recent findings on the functions of Pol IV in plant growth and development and consider other possible functions that need further investigation. 

In plants, RNA-directed DNA methylation (RdDM) is a conserved epigenetic process that mediates the silencing of DNA with repetitive sequences and transposable elements (TEs). Thus, RdDM is considered to be an important mechanism for the maintenance of genome stability (Tsukahara *et al.*, 2009; Law *et al.*, 2010). In the canonical RdDM pathway, RNA-DEPENDENT RNA POLYMERASE 2 (RDR2) converts RNA polymerase IV (Pol IV)-generated transcripts into double-stranded RNAs (dsRNAs), while in the non-canonical RdDM pathway, RNA-DEPENDENT RNA POLYMERASE 6 (RDR6) converts RNA polymerase II (Pol II)-generated RNA transcripts into dsRNAs. The canonical RdDM pathway includes the following steps. (i) SAWADEE HOMEODOMAIN HOMOLOG 1 (SHH1) protein recognizes histone H3K9me2 and then recruits Pol IV to recognize the heterochromatic regions to transcribe precursor RNAs, which are in the order of 25 to 50 nucleotides (nt) in length (Yang *et al.*, 2016). (ii) RDR2 physically interacts with Pol IV (Haag *et al.*, 2012) and converts the Pol IV transcripts into dsRNAs. Among them, the chromatin remodeler proteins CLASSYs (CLSYs; CLSY1–4) function as components of the Pol IV complex; their main function is to act as locus-specific regulators of both 24-nt small interfering RNA (siRNA) production and DNA methylation (Yang *et al.*, 2018; Zhou *et al.*, 2018). (iii) dsRNAs are processed by DICER-LIKE 3 (DCL3) into 24-nt siRNAs, which are loaded into ARGONAUTE4 (AGO4) and then processed through Pol V-mediated *de novo* DNA methylation (Wierzbicki *et al.*, 2008; Zhong *et al.*, 2014; Gallego-Bartolomé *et al.*, 2019; Singh *et al.*, 2019). 

Apart from DCL3, other DCLs are also capable of generating distinct small RNA species. Among them, DCL1 is known to be responsible for the maturation of 21-nt microRNAs (miRNAs) or siRNAs processed from hairpin-structured precursors. DCL2 acts mainly in the biogenesis of 22-nt viral siRNAs (vsiRNAs), while DCL4 generates mainly 21-nt *trans*-acting siRNAs (ta-siRNAs). Furthermore, DCL2, DCL3, and DCL4 are known to function partially redundantly in the establishment and maintenance of DNA methylation as well as the biogenesis of Pol IV-generated RNA transcripts. In addition, there is a unique and DCL-independent class of siRNAs (sidRNAs) of the order of 20 to 60 nt in length (Yang *et al.*, 2016; Ye *et al.*, 2016). The precursor RNA transcripts of sidRNAs are associated with AGO4 and are subsequently trimmed by 3′–5′ exonuclease to produce mature sidRNAs to initiate *de novo* DNA methylation (Ye *et al.*, 2016). Because the RdDM pathway has been found in both vegetative and reproductive organs of plants, it is likely to have prominent roles in the whole life cycle.

## Multifaceted roles of Pol IV in plant growth and development

Very recently, two articles have shed new light on the functions of Pol IV in rice (*Oryza sativa*). Zhang *et al.* (2020) have reported that the stable expression of rice grassy stunt virus (RGSV)-encoded P3 protein in rice plants can cause a dwarfing and excessive tillering phenotype similar to the disease symptoms caused by RGSV infection. The authors conclude that stable expression of P3 protein or RGSV infection in rice plants can lead to an enhancement of ubiquitination and the ubiquitin proteasome system (UPS)-dependent degradation of rice NUCLEAR RNA POLYMERASE D1a (OsNRPD1a), one of the two orthologs of the largest subunit of plant-specific Pol IV holoenzyme. This degradation mechanism is accomplished mainly by recruiting P3IP1, a P3-inducible U-box type E3 ubiquitin ligase, to ubiquitinate and degrade OsNRPD1a protein by the UPS-dependent pathway. This report also revealed that RGSV can target host Pol IV for UPS-dependent degradation and RdDM core protein can serve as a potential target for the UPS, a novel virulence mechanism underlying plant–virus interactions (Zhang *et al.*, 2020).

The other study, by Xu *et al.* (2020), revealed that RdDM inhibits rice tillering by regulating the expression of three agriculturally important genes, *OsMIR156d*, *OsMIR156j*, and *DWARF14* (*D14*). Reduced expression of rice *OsNRPD1a* and *OsNRPD1b* results in a pronounced loss of genome-wide 24-nt siRNAs, a remarkable reduction of DNA methylation in the miniature inverted-repeat transposable element (MITE) regions, especially CHH methylation, and the subsequent control of the expression of key genes associated with rice tillering. Mechanistically, RdDM targets two MITEs in the promoter regions of *OsMIR156d* and *OsMIR156j* and significantly inhibits the transcription of these two miRNAs, which controls the expression of key genes related to rice tillering. Rice tillering determines the plant structure and grain yield, and Ideal Plant Architecture 1 (IPA1) is an important factor that has been identified to regulate rice tillering. Three MITEs were found in the promoter of *IPA1*. However, the degree of methylation of these MITEs was not significantly different between wild-type plants and *osnrpd1-1* mutants. To a certain extent, the possibility of RdDM involvement in the regulation of rice tillering by directly controlling the transcription of *IPA1* was ruled out. Studies have found that the expression of *IPA1* can be inhibited by OsmiR156 at the shoot tip (Jiao *et al.*, 2010; Miura *et al.*, 2010). *OsmiR156a-j* transcripts accumulated excessively in *osnrpd1-1/2* and *osnrpd1ab* double knockout lines, and the expression of the target *IPA1* was down-regulated, highlighting that RdDM regulates rice tillering through the OsmiR156–IPA1 module. In contrast, the expression of *D14*, which encodes a strigolactone receptor and can repress the outgrowth of rice tillers, is activated by CHH methylation in a MITE region located at its downstream. In the *osnrpd1-1/2* mutant, MITE#1 in the downstream region of *D14* was hypomethylated, resulting in the down-regulation of *D14* and enhanced protein stability of D53. Furthermore, D53 inhibits the transcriptional activation ability of *IPA1* (Song *et al.*, 2017), leading to an increase in rice tillering, indicating that RdDM also controls rice tillering through the strigolactone signaling pathway. This finding indicates an important RdDM-dependent mechanism controlling rice tillering and provides potential targets for the improvement of agronomic traits through epigenome editing.

In addition to its above-mentioned roles, Pol IV is also critical for basal heat tolerance in Arabidopsis. Transient heat stress can affect the epigenetic program in plants as well as the long-term thermal responses triggered by the depletion of loci silencing within constitutive heterochromatin. Recent findings have indicated that mutant plants defective in *NRPD2*, which encodes a common (and the second largest) subunit of the Pol IV and Pol V complexes, are hypersensitive to heat exposure. All the dysregulated genes in *nrpd2* mutants recovering from heat stress are located near the transposon residues or the siRNA-producing clusters, suggesting that these dysregulated thermal-responsive genes are modulated by defective epigenetic regulation near the transposons in plants lacking a functional *NRPD2*. These results also point toward a certain signal-controlled correlation between the RdDM pathway and plant tolerance to heat stress (Popova *et al.*, 2013).

Recently, Pol IV has been shown to play an important role in pollen development in Arabidopsis. The formation of pollen is strongly affected by the reprogramming of CHH methylation. During meiosis, the global level of CHH methylation is greatly reduced and the accumulation of meiosis-specific small RNAs is dependent on Pol IV (Walker *et al.*, 2018). Although many functions of Pol IV have been documented, its loss of function does not cause an obvious pollen-deficient phenotype in Arabidopsis. Based on the obvious difference in TE contents between *Arabidopsis thaliana* and *Capsella rubella*, the loss of function of Pol IV has a greater impact on the latter species, resembling the defects in *Brassica rapa* (Grover *et al.*, 2018). Recent studies have also demonstrated that the loss of Pol IV function in *Capsella* can lead to an arrest of microspore development. Small RNA profiling has shown that depletion of Pol IV can block the production of 21- and 22-nt siRNAs (Wang *et al.*, 2020), suggesting that Pol IV is required for the synthesis of epigenetically activated 21- and 22-nt siRNAs (easiRNAs) in pollen. The biogenesis of easiRNAs is known to be triggered by certain miRNAs (e.g. miRNA845b) and requires the involvement of DCL2 and/or DCL4.

Pol IV-dependent paternal easiRNA can cause barriers to cross-breeding using plants of different ploidy (Martinez *et al.*, 2018). Seed development is sensitive to parental genome doses, and excessive paternal genomes can cause defective phenotypes, including large endosperm reproduction without cellularization and seed abortion. Paternal loss of Pol IV function can inhibit easiRNA biogenesis, and depletion of easiRNA can overcome the triploid block to rescue triploid seed formation via the restoration of RdDM on TEs. This restoration will increase paternal ploidy in Arabidopsis. It is noteworthy that easiRNA is not only a quantitative signal for paternal chromosomes, but also a balanced dose required for post-fertilization genome stabilization as well as seed vigor. How easiRNA is generated, and the nature of its downstream reaction mechanisms, are still not fully understood and thus need more in-depth research.

Coinciding with Arabidopsis, the maize (*Zea mays*) Pol IV-mediated RdDM pathway also plays an extensive role in the regulation of genome dominance, subgenome stability, and evolution. Maize *RNA Polymerase D1* (*RPD1*/*RMR6*) encodes the largest subunit of Pol IV, which is necessary for the generation of siRNAs to maintain the gene expression patterns needed for normal plant development. In-depth and comprehensive analyses of gene expression, TE distribution, small RNA targeting, and DNA methylation levels in *rpd1*/*rmr6* mutant plants have shown that the loss of Pol IV activity can result in an overall increase of RNA transcription from the maize genome. Among the regulated genes, those near the TE insertions are the most affected genes via Pol IV-mediated gene silencing, and the TEs in the inserted genes can affect the expression of adjacent loci. The regulation of the expression of nearby genes by TEs is related to the methylation profiles on the flanking regions of the genes and is strictly dependent on the characteristics of the inserted TEs (Erhard *et al.*, 2013; Forestan *et al.*, 2017).

## Future perspectives

**Fig. 1.**
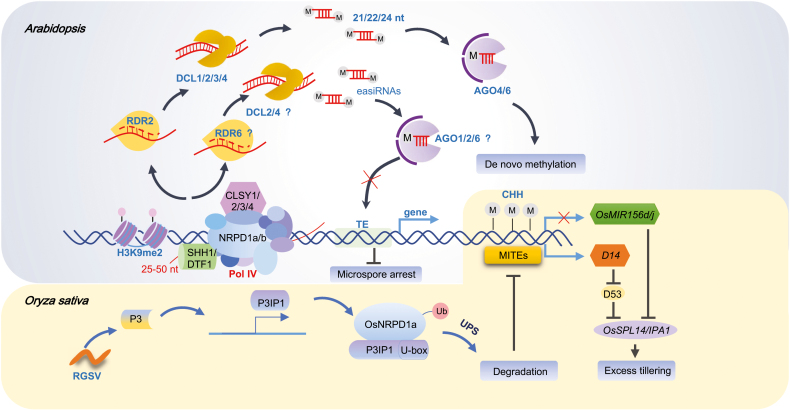
The all-round role of RNA polymerase IV (Pol IV) in plants. In Arabidopsis, SHH1/DTF1 binds to the nucleosome through reading H3K9me2, and recruits Pol IV to transcribe the target region. RDR2 and RDR6 interact with Pol IV to convert and then process the Pol IV transcripts into 21/22/24-nt siRNAs and easiRNAs with the assistance of DCL proteins. Among these, DCL3 is the main enzyme for processing Pol IV-synthesized RNA transcripts, and other DCLs might be more important for easiRNA biosynthesis. As components of the Pol IV complex, the CLSYs (CLSY 1–4) regulate the Pol IV–chromatin association and 24-nt siRNA production at thousands of distinct loci, but whether CLSYs directly bind chromatin is not known. Subsequently, the guide strand is incorporated into AGO4/6, and then enters *de novo* DNA methylation or builds the triploid block using excess 21/22-nt easiRNAs. In rice, depletion of Pol IV (OsNRPD1a and OsNRPD1b) results in a remarkable loss of CHH-type DNA methylation in MITEs, thereby affecting the expression of key agronomically important genes (*OsMIR156d/j* and *D14*) to regulate rice tillering. By recruiting E3 ubiquitin ligase P3IP1, rice grassy stunt virus (RGSV) P3 protein enhances the ubiquitination and UPS-dependent degradation of rice OsNRPD1a. These findings highlight a new virulence mechanism underlying plant–virus interaction, and further integrate the crosstalk between the RdDM pathway and UPS-dependent degradation during virus infection.

A comprehensive list of components associated with Arabidopsis and rice RdDM pathways, including DCLs, AGOs, and RDRs, is given in Table 1. In addition, we have summarized the multifaceted role of Pol IV in plants in Fig. 1. Many recent findings have advanced our knowledge on the functions of Pol IV in five main areas: (i) Pol IV governs the expression pattern of genes near TE insertions to maintain the stability and evolution of maize subgenomes. (ii) Pol IV functions in the reproductive development of crucifer plants (e.g. *C. rubella* and *B. rapa*). (iii) Pol IV functions in monocot plant (e.g. *O. sativa*) morphogenesis. (iv) Pol IV functions in the barriers that arise during plant cross-breeding. (v) Pol IV participates in the regulation of plant resistance to abiotic and biotic stresses.

**Table 1.** RdDM components and other DNA methylation-related factors in plants

Protein components involved in the RdDM pathway and DNA methylation in Arabidopsis			
Proteins	Gene ID	Description	Reference
AtNRPD1a	*At1G63020*	One of the two alternative largest subunits of Pol IV	(Luo and Hall, 2007)
AtNRPD1b/AtNRPE1	*At2G40030*	Unique largest subunit of Pol V	(Wendte *et al.*, 2017)
AtNRPD2a	*At3G23780*	Shared, the second largest catalytic subunit of Pol IV	(Herr *et al.*, 2005)
AtNRPD4/AtNRPE4	*At4G15950*	Non-catalytic subunit of Pol IV and Pol V	(He *et al.*, 2009a)
AtNRPE5	*At3G57080*	Non-catalytic subunit of Pol V	(Eun *et al.*, 2012)
AtNRPE9b	*At4G16265*	One of the two highly similar non-catalytic subunits of Pol II, Pol IV, and Pol V	(Tan *et al.*, 2012)
AtRDR2	*At4G11130*	RNA-dependent RNA polymerase acting together with Pol IV	(Haag and Pikaard, 2011)
AtRDR6	*At3g49500*	RNA-dependent RNA polymerase acting together with Pol II	(Nuthikattu *et al.*, 2013)
AtDCL1	*At1g01040*	Dicer endonuclease that generates 21/22 nt miRNAs	(Zhang *et al.*, 2018)
AtDCL2	*At3G03300*	Dicer endonuclease that generates 22 nt siRNAs	(Stroud *et al.*, 2013)
AtDCL3	*At3G43920*	Dicer endonuclease that generates 24 nt siRNAs	(Wei *et al.*, 2014)
AtDCL4	*At5g20320*	Dicer endonuclease that generates 21 nt tasiRNAs	(Stroud *et al.*, 2013)
AtHEN1	*At4G20910*	RNA methyltransferase	(Baranauskė *et al.*, 2015)
AtAGO1	*At1G48410*	Initiates *de novo* DNA methylation through the RDR6–RdDM pathway	(Kenesi *et al.*, 2017)
AtAGO4	*At2G27040*	Argonaute protein in the AGO4 clade, specialized for the RdDM pathway.	(Pikaard *et al*., 2012)
AtAGO6	*At2G32940*	Argonaute protein in the AGO4 clade, specialized for the RdDM pathway.	(Bologna and Voinnet, 2014)
AtAGO7	*At1G69440*	Involved in the regulation of developmental timing	(Qu *et al.*, 2008)
AtAGO9	*At5G21150*	Argonaute protein in the AGO4 clade, role in RdDM uncertain	(Bologna and Voinnet, 2014)
AtDMS3	*At3G49250*	Facilitates RNA1-mediated epigenetic modification, involving secondary siRNA production and spreading of DNA methylation	(Law *et al.*, 2010)
AtRDM1	*At3G22680*	AGO4- and Pol II-interacting protein	(Law *et al.*, 2010)
AtDMS4/AtRDM4	*At2G30280*	Putative nuclear import factor for Pol II, Pol IV, and Pol V	(He *et al.*, 2009b)
AtSPT5-like	*At5G04290*	Contains an AGO hook motif, involved in *Pol V* transcription	(Hartzog and Fu, 2013)
AtIDN2	*At3G48670*	dsRNA-binding protein in the Pol V pathway	(Ausin *et al.*, 2009)
AtIDP1	*At1G15910*	Forms a complex with IDN2	(Zhang *et al.*, 2012)
AtIDP2	*At4G00380*	Forms a complex with IDN2	(Zhang *et al.*, 2012)
AtSWI3B	*At2G33610*	Subunit of the SWI/SNF chromatin-remodeling complex	(Jie *et al.*, 2020)
AtDRM2	*At5G14620*	*de novo* DNA methyltransferase	(Henderson *et al.*, 2010)
AtSUVH2	*At2G33290*	SRA domain protein that binds to methylated DNA and recruits Pol V	(Kuhlmann and Mette, 2012)
AtSUVH4	*At5G13960*	H3K9 methyltransferase	(Du *et al.*, 2014)
AtSUVH9	*At4G13460*	SRA domain protein that binds to methylated DNA and recruits Pol V	(Kuhlmann and Mette, 2012)
AtSHH1	*At1G15215*	An atypical RNA-directed DNA methylation component	(Law *et al.*, 2013)
AtHDA6	*At5G63110*	Histone deacetylase	(Aufsatz *et al.*, 2002)
AtJMJ14	*At4G20400*	Histone demethylase	(Searle *et al.*, 2010)
AtUBP26	*At3G49600*	Histone H2B deubiquitinase	(Sridhar *et al.*, 2007)
AtNERD	*At2G16485*	Involved in the non-canonical RdDM pathway	(Pontier *et al.*, 2012)
AtCMT2	*At4G19020*	DNA methyltransferase specific for CHH	(Zemach *et al.*, 2013)
AtCMT3	*At1G69770*	DNA methyltransferase specific for CHG	(Cao *et al.*, 2003)
AtMET1	*At5G49160*	DNA methyltransferase specific for CG	(Pikaard and Scheid, 2014)
AtDDM1	*At5G66750*	Snf2 chromatin remodeler acting in siRNA-independent DNA methylation	(Zemach *et al.*, 2013)
AtDRD1	*At2G16390*	Putative Snf2 chromatin remodeling factor, part of the DDR complex; involved in the Pol V pathway	(Kanno *et al.*, 2004)
AtCLSY1	*At3G42670*	Putative Snf2 chromatin remodeling factor, involved in the Pol IV pathway	(Smith *et al.*, 2007)
AtCHR34	*At2G21450*	Putative Snf2 chromatin remodeling factor	(Kanno *et al.*, 2004)
AtCLSY2	*At5G20420*	Putative Snf2 chromatin remodeling factor, involved in the Pol IV pathway	(Law *et al.*, 2011)
AtCLSY3	*At1G05490*	Putative Snf2 chromatin remodeling factor, involved in the Pol IV pathway	(Law *et al.*, 2011)
AtCLSY4	*At3G24340*	Putative Snf2 chromatin remodeling factor, involved in the Pol IV pathway	(Law *et al.*, 2011)
AtMORC1	*At4G36290*	GHKL-type ATPase	(Moissiard *et al.*, 2012)
AtMORC2	*At4G36280*	GHKL-type ATPase	(Kang *et al.*, 2012)
AtMORC3	*At4G36270*	GHKL-type ATPase	(Harris *et al.*, 2016)
AtMORC4	*At5G50780*	GHKL-type ATPase	(Harris *et al.*, 2016)
AtMORC5	*At5G13130*	GHKL-type ATPase	(Koch *et al.*, 2017)
AtMORC6	*At1G19100*	GHKL-type ATPase	(Brabbs *et al.*, 2013)
AtMORC7	*At4G24970*	GHKL-type ATPase	(Harris *et al.*, 2016)
AtSPT5-1	*At2G34210*	Transcription elongation factor	(Dürr *et al.*, 2014)
AtSPT5-2	*At4G08350*	Transcription elongation factor	(Dürr *et al.*, 2014)
AtNRPB1	*At4G35800*	Largest subunit of Pol II	(Haag and Pikaard, 2011)
AtNRPB2	*At4G21710*	Second largest subunit of Pol II	(Onodera *et al.*, 2005)
AtROS1	*At2G36490*	DNA glycosylase/lyase acting in active demethylation of DNA	(Zhang and Zhu, 2012)
AtGMI1	At5G24280	DNA double-strand break repair	(Böhmdorfer *et al.*, 2011)
Protein components involved in the RdDM pathway and DNA methylation in rice			
OsNRPD1a	*LOC_Os04g48370*	One of two orthologs of the largest subunit of Pol IV	(Zhang *et al.*, 2020)
OsNRPD1b	*LOC_Os09g38268*	One of two orthologs of the largest subunit of Pol IV	(Xu *et al.*, 2020)
OsDCL1a	*LOC_Os03g02970*	Responsible for the processing of 21/24-nt miRNAs	(Liu *et al.*, 2005)
OsDCL2a	*LOC_Os03g38740*	Responsible for the processing of rice miRNAs	(Kapoor *et al.*, 2008)
OsDCL2b	*LOC_Os09g14610*	Responsible for the processing of rice miRNAs	(Kapoor *et al.*, 2008)
OsDCL3a	*LOC_Os01g68120*	Required for the biogenesis of lmiRNAs	(Kapoor *et al.*, 2008)
OsDCL3b	*LOC_Os10g34430*	Responsible for the processing of 21/24-nt miRNAs	(Song *et al.*, 2012)
OsDCL4	*LOC_Os04g43050*	Affects the production of 21nt siRNA in the panicle	(Song *et al.*, 2012)
OsAGO1a	*LOC_Os02g45070*	Has the ability to bind small RNA and has cleavage activity	(Wu *et al.*, 2009)
OsAGO1b	*LOC_Os04g47870*	Has the ability to bind small RNA and has cleavage activity	(Wu *et al.*, 2009)
OsAGO1c	*LOC_Os02g58490*	Has the ability to bind small RNA and has cleavage activity	(Wu *et al.*, 2009)
OsAGO1d	*LOC_Os06g51310*	Member of RNA-induced silencing complex	(Wu *et al.*, 2009)
OsAGO2	*LOC_Os04g52540*	Involved in DNA methylation, active oxygen metabolism regulation, tapetum development, and programmed cell death	(Zheng *et al.*, 2019)
OsAGO4a	*LOC_Os01g16870*	Involved in the biogenesis of small RNAs	(Kapoor *et al.*, 2008)
OsAGO4b	*LOC_Os04g06770*	Involved in the biogenesis of small RNAs	(Kapoor *et al.*, 2008)
OsMEL1	*LOC_Os03g58600*	Participates in the regulation of the division of germ cells before meiosis, the correct modification of meiotic chromosomes, and the accurate progress of meiosis through the RdDM pathway	(Nonomura *et al.*, 2007)
OsAGO16	*LOC_Os07g16224*	Involved in transcriptional gene silencing by guiding DNA methylation	(Wu *et al.*, 2010)
OsSHL4/OsAGO7	*LOC_Os03g33650*	Affects the development of leaf polarity	(Itoh *et al.*, 2000)
OsPNH1	*LOC_Os06g39640*	Regulates apical meristems, vascular bundle development, and leaf formation	(Nishimura *et al.*, 2002)
OsAGO17	*LOC_Os02g07310*	Has crucial regulatory roles in rice pollen development	(Yao *et al.*, 2018)
OsAGO18	*LOC_Os07g28850*	Confers broad-spectrum virus resistance in rice	(Wu *et al.*, 2015)
OsSHL2/OsRDR6	*LOC_Os01g34350*	Participates in the plant defense responses to viruses, bacteria, and fungi	(Wagh *et al.*, 2016)
OsRDR2	*LOC_Os04g39160*	Has roles in siRNA-mediated DNA methylation and histone modifications	(Vrbsky *et al.*, 2010)
OsRDR4	*LOC_Os01g10140*	Specifically activated in response to dehydration stress	(Kumar and Singh, 2016)
OsRDR1	*LOC_Os02g50330*	Involved in the antiviral RNA silencing pathway	(Wang *et al.*, 2016)
OsRDR3	*LOC_Os01g10130*	Specifically activated in response to dehydration stress	(Kumar and Singh, 2016)
OsCMT3	*LOC_Os10g01570*	Involved in the epigenetic process affecting genome activity during abiotic stress	(Sharma *et al.*, 2009)
OsCMT2	*LOC_Os05g13790*	Has a role in CHH methylation	(Lanciano and Mirouze, 2017)
OsMET1-1	*LOC_Os03g58400*	Has a minor and/or redundant role in maintaining the CG methylation	(Lanciano and Mirouze, 2017)
OsMET1-2	*LOC_Os07g08500*	Has critical roles in maintaining ^m^CG in rice	(Hu *et al.*, 2020)
OsDRM2	*LOC_Os03g02010*	Regulates rice vegetative and reproductive growth through DNA methylation	(Dangwal *et al.*, 2013)

Although many studies using Arabidopsis, rice, maize, and other plants have significantly advanced our knowledge on the functions of Pol IV, many fundamental questions are still unanswered. For example, Pol IV is an important component in RdDM, and rice *nrpd1* mutant plants exhibit a dwarfed and excessive tillering phenotype, and maize *rpd1* mutants are shorter, with delayed flowering, feminization of male tassels, depolarization of leaf tissue, and tissue outgrowths on their stems (Parkinson *et al.*, 2007, Erhard *et al.*, 2009). In contrast to these representative monocotyledonous species, Arabidopsis mutants in Pol IV function have no such developmental defects. Perhaps Pol IV controls different regulatory mechanisms in monocotyledonous and dicotyledonous plants. As far as monocotyledonous species are concerned, the loss of Pol IV activity also has different effects on plant development in rice and maize, and its underlying fine mechanisms still need to be urgently elucidated in future research. In addition, it remains unknown how RGSV can target host Pol IV to disrupt the UPS-dependent pathways but not the downstream regulatory networks involved in plant–pathogen interactions. Although OsNRPD1a and OsNRPD1b are the orthologs of the largest subunit in rice Pol IV, do they have functional divergence, especially in the regulation of plant responses to stresses? Can Pol IV play roles in other abiotic stress responses in addition to heat stress,?

Future biochemical, functional, and genetic studies are necessary to address these questions. As with other molecular biology studies, the studies on the functions of Pol IV have entered a new phase to explore much broader and more in-depth mechanisms in many other plant species. Understanding the mechanisms underlying the functions of Pol IV in other plant species, especially monocotyledonous species, will provide us with opportunities to identify the links between RdDM and other molecular pathways, such as the UPS-dependent pathway. Collectively, the information described above will uncover the multifaceted roles of Pol IV in plant development and reproduction.

## Data Availability

All data supporting the findings of this viewpoint are available from the corresponding author, Jian-Guo Wu, upon request.
